# Diagnosis and management of Neuro-Behçet disease with isolated intracranial hypertension: a case report and literature review

**DOI:** 10.1186/s12883-023-03392-3

**Published:** 2023-09-25

**Authors:** Yali Wu, Wei Yin, Shufang Liu, Shasha Wang, Yan Ding

**Affiliations:** 1 Department of Rheumatology and Immunology, Wuhan Children’s Hospital (Wuhan Maternal and Child Healthcare Hospital), Tongji Medical College, Huazhong University of Science & Technology, Wuhan, 430016 China; 2 Department of Ophthalmology, Wuhan Children’s Hospital (Wuhan Maternal and Child Healthcare Hospital), Tongji Medical College, Huazhong University of Science & Technology, Wuhan, 430016 China

**Keywords:** Behçet's disease, Intracranial hypertension, Papilledema, Neuro-Behçet’s disease

## Abstract

**Background:**

Neuro-Behçet's disease (NBD), characterized by isolated intracranial hypertension, is a rarely encountered condition, especially in children. In this study, we describe the treatment of a pediatric patient with NBD, who exhibited isolated intracranial pressure elevation as indicated by the initial manifestation of diplopia and physical examination finding of papilledema.

**Case presentation:**

An 8-year-old boy was admitted to the hospital with a confirmed diagnosis of Behçet's disease (BD) over eight months. The patient also presented with the symptom of diplopia for three days. The evaluation of the patient’s nervous system did not reveal any apparent abnormalities. The measurement of cerebrospinal fluid pressure yielded a reading of 470 mm H_2_O. The examination of the fundus indicated papilledema, and imaging scans revealed evidence of focal demyelination. The symptoms of the child showed alleviation after the administration of mannitol, methylprednisolone, and azathioprine. Furthermore, this study involved a comprehensive analysis of 18 cases of NBD with isolated intracranial hypertension, comprising one case reported herein and 17 cases from the literature review. Three cases were children, and an equal distribution of males and females 9:9 was noted. The average age at the onset of symptoms was 24.7 years (8-38 years). Headache (90%) was the most commonly reported clinical manifestation, followed by blurred vision or diplopia (80%). The ocular manifestations included papilledema (100%), abducent nerve paralysis (20%) and local eye hemorrhages in the retina (30%). Notably, 88.9% of these ocular manifestations were relieved or cured after treatment.

**Conclusion:**

This study presents the first reported case of NBD with isolated intracranial hypertension in the pediatric population of China. In a child with Bechet's disease presenting with features of raised intracranial pressure, it is important to be aware of neuro Bechet's presenting with intracranial hypertension without other neurological abnormalities. This will help make early diagnosis, institute treatment and prevent sequelae resulting from untreated raised intracranial pressure.

## Background

Behçet's disease (BD) is a vasculitic disorder characterized by recurrent chronic involvement of multiple systems, which is triggered by infection and environmental factors in a specific genetic background [1, 2]. This disease primarily affects the skin, mucous membranes, and various other organs. Typical clinical manifestations include recurrent oral ulcers, genital ulcers, and uveitis, which may lead to skin damage and arterial and venous thrombosis. Other systems can also be affected, including the gastrointestinal tract, nerves, cardiovascular system, respiratory system, and urinary system. Notably, the involvement of the blood system can be complicated by myelodysplastic syndrome [3]. The presence of neurological complications in BD is referred to as neuro-Behçet's disease (NBD), which can cause adverse outcomes such as disability or even death [4]. The precise incidence of the disease is unclear; however, it is estimated that less than 10% of patients with BD will develop NBD [5, 6]. Moreover, the occurrence of NBD in children is rarely reported. This study retrospectively analyzed the clinical features of NBD with isolated intracranial hypertension as the primary manifestation in the first reported pediatric case in China. Notably, this case exhibited no brain parenchyma damage or venous sinus thrombosis. Furthermore, a comprehensive review of the existing literature was conducted to summarize the clinical features, treatment modalities, and prognostic outcomes of NBD.

## Case presentation

### History

An 8-year-old boy was admitted to the hospital after having received a diagnosis of BD for eight months. His initial hospital admission occurred due to complaints of pain in both lower limbs for half a month. The patient was diagnosed with BD based on the manifestation of recurrent oral ulcers, there were pustular cutaneous lesions at the site of acupuncture, venous thrombosis in both lower limbs, arthritis, multiple ulcers in the duodenal bulb, end of ileum, colon, and rectum, and the identification of vasculitis in tissue biopsy from the end of the ileum and ileocecal valve. Following treatment, the patient was discharged from the hospital in a stable state and was advised to maintain the prescribed medications, including prednisone, cyclosporine, thalidomide, and others, to manage the primary disease. During subsequent visits to the outpatient department, the patient’s hormone dosage would be gradually reduced, as discussed and reviewed. The discontinuation of prednisone and cyclosporine occurred approximately two months ago, with methotrexate and thalidomide introduced as alternative therapeutic interventions to manage the patient’s conditions. However, in this circumstance, the patient presented with diplopia for three days, accompanied by vomiting with intermittent headaches for one day, which prompted their admission to the hospital for further assessment and treatment.

### Physical examination and data collection

General physical examination showed no neck resistance or other observable positive signs related to the nervous system. Additionally, there were no oral or vulvar ulcers observed, nor were there any noticeable erythema folliculitis-like changes. Neurological examination showed clear consciousness, facial symmetry, the fundus examination revealed papilledema in both eyes, along with slightly tortuous retinal blood vessels and orthophoria. After alternate covering, a slight limitation in the outward rotation was detected in the left eye. However, all other parameters, including visual acuity, intraocular pressure, and slit lamp tests, indicated normal findings. Tone, muscle strength, reflexes, sensory system, cerebellar signs, meningeal signs, or involuntary movements are negative. Systemic examination showed blood routine analysis, C-reactive protein (CRP) level, erythrocyte sedimentation rate, ferritin levels, comprehensive biochemistry panel, assessment of cellular immunity, evaluation of humoral immune function, screening for tuberculosis, fungi, parasites, chronic viruses, and other pathogens, arteriovenous ultrasound of the limbs, and chest and abdomen computed tomography (CT) scans, returned normal results. Brain magnetic resonance imaging (MRI), magnetic resonance angiography (MRA), and magnetic resonance venography (MRV) showed abnormal focal signal changes in both frontal and parietal lobes representing focal demyelination, with no other abnormalities observed. The MRI scans of both eyes provided normal results. The cerebrospinal fluid (CSF) opening pressure was measured at 470 mmH_2_0, while the chloride ion concentration in the biochemical analysis of CSF was 129 mmol/L. Other CSF examinations, including protein, glucose, cells in CSF, acid-fast staining, ink staining, and etiological tests, yielded normal outcomes.

### Diagnosis and treatments

After the patient’s admission, the initial medication regimen was maintained, with the prompt administration of mannitol. A comprehensive consultation involving the departments of ophthalmology, neurology, imaging, and rheumatology and immunology, was conducted following the receipt of laboratory tests and cranial imaging results. Through a multidisciplinary meeting involving expertise of specialties in ophthalmology, neurology, imageology, pharmacology, immunology and rheumatology, a consensus diagnosis of NBD, intracranial hypertension, bilateral papilledema, and left ocular abductor insufficiency paralysis was established. After the therapeutic intervention, the administration of methotrexate and thalidomide was discontinued, and methylprednisolone (2 mg/kg/day), azathioprine (50 mg/day), mannitol, and mecobalamin were introduced. After eight days, the symptoms of diplopia, headache, and vomiting were alleviated, accompanied by a restoration of the intracranial pressure to its normal range. After four weeks, a fundus examination revealed no obvious papilledema.

### Clinical manifestations

**Fig. 1:**
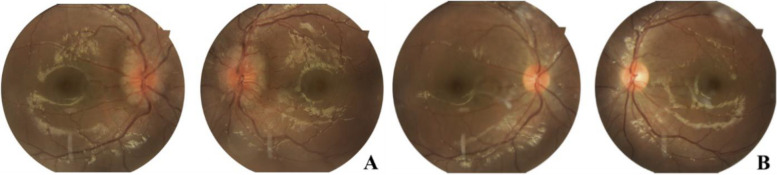
Representative images of fundoscopic examinations. Panel **A**: Initial assessment showing papilledema in both eyes, along with slightly tortuous retinal vessels. Panel **B**: Significant improvement in papilledema and retinal vascular tortuosity after two weeks of treatment

**Fig. 2:**
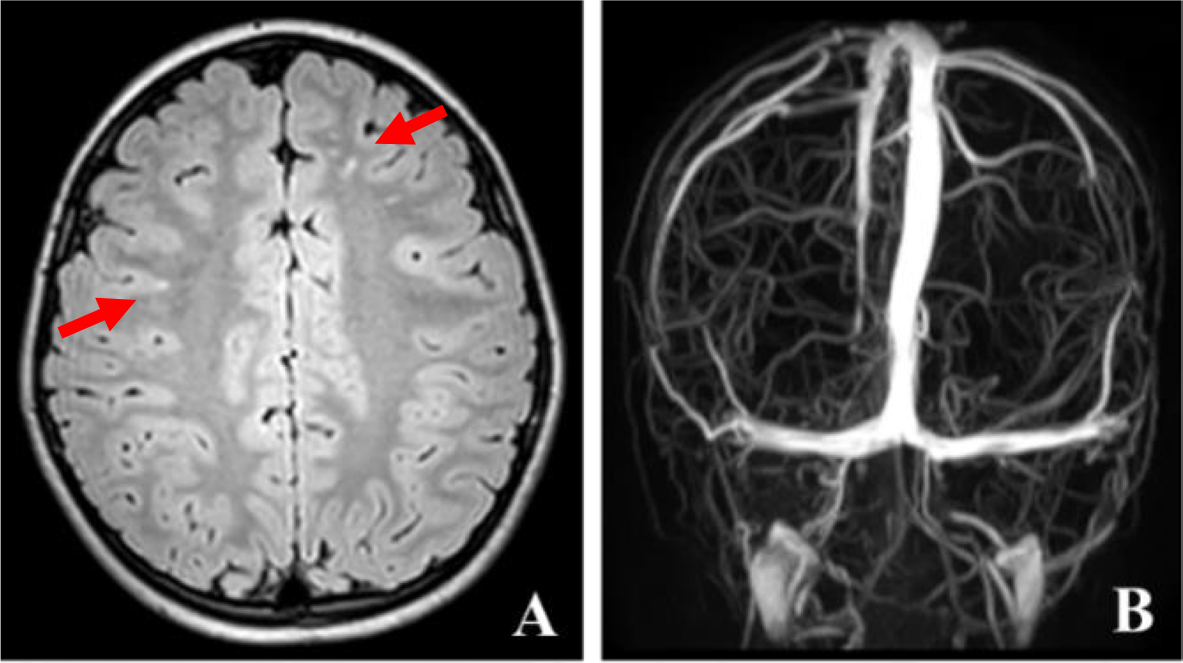
Representative scanning images of the nervous system. Panel **A**: MRI image of the patient's brain. Deep left frontal lobe and the right parietal lobe showing high signal intensity at T2 FLAIR imaging, as indicated by the arrows. Panel **B**: MRV image of the patient's brain without noticeable abnormalities. MRI, magnetic resonance imaging; MRV, magnetic resonance venography

This case report presents the clinical findings of an 8-year-old boy with an insidious onset of symptoms. The primary complaints included diplopia for three days, accompanied by headache and vomiting for one day. The examination revealed obvious signs of papilledema, isolated raised intracranial pressure without any damage to the brain parenchyma, and venous sinus thrombosis. No other disorders, such as craniocerebral injury, space-occupying lesions, infections, hypoxia, or electrolyte abnormalities, were detected. The final diagnosis was confirmed as NBD, intracranial hypertension, bilateral optic disc edema, and abductor insufficiency paralysis. The administration of medications resulted in significant improvements in the patient's condition. The detailed examinations of the eye and nervous system are depicted in Figs. 1 and 2.

## Literature review

A comprehensive literature review was conducted by searching the PubMed database was searched from its inception to June 2022. The search yielded a total of 107 articles using the keywords “Behcet's disease” and “intracranial hypertension,” and 74 articles using the keywords “Behcet's disease” and “papilledema.” After the removal of duplicate articles, 58 articles were included, and the full texts were carefully read. Through this selection, six articles were included, which collectively reported 17 patients (including children and adults). The subsequent analysis integrated the 17 cases from the literature with the current case study, and the clinical data were extracted and summarized.

The six articles selected through the literature review involved 17 patients. With the inclusion of the current case, the total number of patients with NBD presenting with isolated intracranial pressure as the primary manifestation reached 18. Tables 1 and 2 provides the details of general conditions, clinical manifestations, test results, treatment, and other information of all patients, accompanied by incidence statistics. Table 3 summarizes the aforementioned incidence data.

**Table 1:** General situation and clinical manifestations associated with isolated intracranial hypertension in patients with NBD

**Article**	**Age**	**Sex**	**Onset symptom**	**Eye symptoms**				**BD symptoms**						
				P	S	Others		M	G	U	SL	PT	A	ST
Teh LS, *et al*. [7]/1990	30 y	M	Headache	**+**				**+**	**+**				**+**	**+**
	36 y	F	Headache and blurred vision	**+**	**+**	Fundus hemorrhage and loss of vision		**+**	**+**				**+**	
Pamir, *et al*. [8]/1981	32 y	F	Headache and blurred vision	**+**				**+**	**+**	**+**		**+**	**+**	
	22 y	M	Headache and blurred vision	**+**		Vitreous hemorrhage		**+**	**+**					**+**
	14 y	M	Headache and fatigue	**+**				**+**					**+**	
Wilkins, *et al*. [9]/1986	23 y	M	Headache, nausea, and blurred vision	**+**				**+**	**+**					
	32 y	F	Headache, vomiting, and blurred vision	**+**				**+**	**+**		**+**			
Lin, *et al*. [10]/2021	38 y	M	Blurred vision and sparkling	**+**		Left visual field defect		**+**	**+**		**+**		**+**	
Neudorf, *et al*. [11]/2003	12 y	F	Headache and diplopia	**+**		Retinal hemorrhage		**+**	**+**					
Akdal, *et al*. [12]/2017	26-57 y	M 3; F 5	N	**+**				**+**	**+**					
Current report	8 y	M	Headache, vomiting, and diplopia	**+**	**+**			**+**			**+**	**+**		**+**

*NBD* Neuro-Behcet’s disease, *y* year, *BD* Behcet’s disease, *F* Female, *M* Male, *N* Not given, *P* Papilledema, *S* Sixth nerve palsy, *M* Mouth ulcer, *G* Genital ulcer, *U* Uveitis, *SL* Skin lesion, *PT* Pathergy test, *A* Arthritis/arthralgia, *ST* Superficial thrombophlebitis

**Table 2:** Findings, treatment, and prognosis associated with isolated intracranial hypertension in patients with NBD

**Article**	**Lumbar CSF pressure (mmH**_**2**_**O)**	**Routine and biochemical tests of CSF**	**Brain imaging**	**Inflammatory markers**	**Treatment**	**Prognosis**
Teh LS, *et al*. [7]/1990	>300	-	-	N	GCS, Aza	Remission in 1 month, recurrence after 6 months, and final recovery
	>300	-	-	N	GCS, Aza	Remission after 1 week, posterior optic atrophy
Pamir, *et al*. [8]/1981	300	Pro 20 mg/dl	N	ESR 81	GCS, Ac	Recovery after 4 months
	300	Pro 30 mg/dl	-	ESR 50	GCS, Ac, Bitemporal decompression.	Secondary optic atrophy after 1 year
	260	108 mg/dl	Encephaledema	N	GCS, Ac	Remission after 2 months
Wilkins, *et al*. [9]/1986	Raised	-	-	ESR 33	Chlorambucil	Remission
	320	-	-	N	GCS, Chlorambucil	Remission, relapse after 8 months, and recovery
Lin, *et al*. [10]/2021	354	-	-	N	GCS, Ac, Aza	Remission after 6 months
Neudorf, *et al*. [11]/2003	500	-	-	N	GCS, Ac→Colchicine	Recovery
Akdal, *et al*. [10]/2017	>250	2 cases WBC >5	-	N	GCS, Ac, Aza; 1 case of optic nerve sheath fenestration	1 case of progression of irregular medication to parenchyma injury, remission in others
Current report	460	-	-	-	GCS, Az, Dehydrator	8-day remission

*NBD* Neuro-Behcet’s disease, *CSF* Cerebrospinal fluid, “-” Negative, *Pro* Protein, *N* Not given, *GCS* Glucocorticoid, *Aza* Azathioprine, *Ac*, Acetazolamide

**Table 3:** Proportion of isolated intracranial hypertension in patients with NBD

**Category**		**Proportion**	**Percentage**
**General**	Male: Female	9:9	
	Average age	24.7 (8-38) y	
**Clinical manifestations**	Headache	9/10	90%
	Blurred or diplopia	8/10	80%
	Nausea/vomiting	3/10	30%
	Papilledema	18/18	100%
	Abducens nerve palsy	2/10	20%
	Eye hemorrhage	3/10	30%
	Oral ulcer	18/18	100%
	Genital ulcer	16/18	88.9%
	Uveitis	1/10	10%
	Skin manifestations	3/10	30%
	Arthritis/arthralgia	2/10	20%
	Superficial thrombophlebitis	3/10	30%
	Lumbar CSF pressure ≥ 300 mmH_2_O	8/10	80%
	Glucocorticoid	18/18	100%
**Treatment**	Dehydrator	14/18	77.8%
	Azathioprine	12/18	66.7%
	Remission/recovery	16/18	88.9%
**Prognosis**	Disease recurrence	2/18	11.1%
	Optic atrophy	2/18	11.8%
	Parenchyma injury	1/18	5.6%

*NBD* Neuro-Behcet’s disease, *y* Year

## Discussion and conclusion

NBD commonly occurs within five years after the diagnosis of BD. The incidence of NBD in males is approximately three times higher than in females [4], with notable impacts on the central and peripheral nervous systems. Based on the localization of manifestations, NBD can be classified into different categories, including parenchymal, non-parenchymal, mixed, and peripheral nervous system involvement. The parenchymal injury is the most prevalent (approximately 80%) form of neurological damage, serving as a leading contributor to disability and death [13]. This subtype predominantly affects the diencephalon, brainstem, basal ganglia, spinal cord, and cerebral hemisphere [14]. On the other hand, non-parenchymal injury, also known as vascular type, accounts for about 10-20% of all cases o^[[6]]^, mainly causing cerebral venous sinus thrombosis, intracranial hypertension (or pseudotumor-like elevated intracranial pressure), intracranial or external carotid aneurysm, acute meningeal syndrome, and others related manifestations [15]. Among these manifestations, venous sinus thrombosis is the most prevalent, typically characterized by severe headache, vomiting, and even disturbance of consciousness lasting from several days to weeks [16]. Ophthalmological examination often reveals papilledema symptoms and occasionally abducens nerve palsy. The term “mixed” refers to the simultaneous occurrence of both parenchymal and non-parenchymal manifestations. The peripheral nervous system may present with isolated cranial and sensory nerve involvement, including the abducens or oculomotor nerves [17, 18]. Disorders of the abducens nerve may be secondary to manifestations of intracranial hypertension, while oculomotor abnormalities are classified as polyneuropathy or autonomic neuropathy [18]. However, the incidence of mixed and peripheral neuropathy is rare.

The patient, an 8-year-old boy, presented with diplopia, elevated CSF pressure of 470 mmH_2_0, obvious papilledema, and abducens insufficiency palsy, all indicative of elevated intracranial pressure. Notably, the patient exhibited no oral ulcers, vulvar ulcers, uveitis, nodular erythema, or abnormal levels of inflammatory markers. Analysis of the brain MRI revealed a few focal demyelinating changes, but the patient did not display any abnormal brain functions, such as unconsciousness and convulsions. Moreover, the MRI findings revealed no typical involvement of the thalamus, brainstem, spinal cord, or other brain parenchyma regions, and provided no evidence of venous sinus thrombosis or meningitis. To summarize, the underlying cause of intracranial hypertension in this pediatric case does not appear to be attributed to tumors, infections, or other connective tissue diseases. Consequently, a team of multidisciplinary experts has diagnosed the patient with NBD, characterized by isolated non-parenchymal raised intracranial pressure.

Currently, cases of NBD with isolated intracranial hypertension are rare, and the clinical characteristics remain unclear. This study summarizes and evaluates the clinical characteristics of 18 reported cases. Interestingly, an equal prevalence has been observed between men and women. The age of onset is relatively young, with an average age of 24.7 years old. The reported cases span from the youngest patient at eight years old to the oldest at 57 years old. The primary clinical manifestations include headache (90%), blurred vision or diplopia (80%), nausea, and vomiting (30%). Thus, it is advised that patients diagnosed with BD who experience these symptoms should promptly seek consultation with an ophthalmologist and undergo cranial MRI and CSF examination simultaneously. All patients with elevated intracranial pressure were all complicated by papilledema. Among these patients, 80% demonstrated a significant increase in intracranial pressure of ≥ 300 mmH_2_O, about 20% had abducens palsy, and 30% displayed fundus and retinal hemorrhages. Isolated intracranial hypertension can emerge in the active stage of BD or months to years after achieving disease control [12]. About 70% of patients with BD can be complicated with ocular involvement, with uveitis being the most common [19]. In this study, it was observed that out of the 18 patients included, all exhibited papilledema, and some had abducens nerve involvement. However, only one patient had a prior diagnosis of uveal disease. Therefore, it can be concluded that individuals with NBD and isolated intracranial hypertension may have a lower risk of uveitis. The causes of papilledema and abducens nerve involvement were found to be associated with increased intracranial pressure or vasculitis, while abducens nerve palsy may also be associated with inflammation of peripheral nerves. It is recommended that patients with BD should adhere to a schedule of regular ophthalmic outpatient examinations. In cases where papilledema is identified, prompt brain imaging and CSF examinations are advised. If significant increases in intracranial pressure are detected, immediate treatment initiation is crucial, along with consistent monitoring. Failure to address these conditions in a timely manner may lead to deterioration of visual acuity, optic nerve atrophy or other adverse consequences [20].

In relation to existing literature, the clinical manifestations of intracranial hypertension and the markedly elevated CSF pressure, in this case, are basically consistent. However, the distinctive aspect lies in the early onset of the disease with peripheral nerve involvement at a young age. Notably, this pediatric case is the first documented case in China and the youngest in the world.

For the diagnosis of NBD with isolated intracranial hypertension, certain conditions related to cerebral pseudotumor syndrome must be met in accordance with the diagnostic criteria of BD. These conditions include: 1. bilateral papilledema; 2. lumbar CSF pressure >250 mmH_2_O; 3. no mass lesions or hydrocephalus on brain imaging [12]. Except for one patient who did not provide specific CSF pressure information, the remaining 17 patients met the diagnostic criteria. The underlying pathogenesis of NBD with isolated hypertension may be linked to immune-mediated impairment of CSF absorption by arachnoid villi or mild inflammation in the venous sinus wall, which may not be identified on contrast-enhanced MRI images [21]. Particularly, isolated intracranial hypertension in BD may not be accompanied by systemic hyperinflammatory response or elevated levels of inflammatory markers, such as erythrocyte sedimentation rate, C-reactive protein, and cytokines [15]. Moreover, the underlying cause for this observation is largely unknown. One possible explanation is that local inflammation may not be sufficient to trigger a systemic inflammatory response. Hence, it is crucial to conduct regular assessments of routine indicators and to prioritize eye and nervous system examinations for early diagnosis and treatment. Glucocorticoids, immunosuppressants, and dehydrating agents are commonly used in the treatment of NBD with isolated intracranial hypertension. The immunosuppressant of choice is azathioprine, which is consistent with the findings of previous reports [13]. However, before initiating its usage, it is important to consider the presence of relevant genes associated with azathioprine methyltransferase activity. For patients with refractory intracranial hypertension or severe visual impairments, fenestration of the optic nerve sheath can be considered for decompression. Early diagnosis and treatment contribute to a favorable prognosis, with clinical relief or complete recovery witnessed in 88.9% of patients. Nevertheless, two patients developed optic atrophy, and two experienced recurring episodes of elevated intracranial pressure despite initial improvement. In our pediatric case of NBD with isolated intracranial hypertension, significant improvement was achieved through the administration of corticosteroids, dehydrating agents, and immunosuppressive agents.

During our analysis of the factors contributing to the elevated intracranial pressure in the child with BD, our team carefully considered the potential influence of the medications being administered. The child had been prescribed oral methotrexate and thalidomide to control the disease for 2 months prior to the onset of symptoms. Upon reviewing the drug inserts for both medications, we found no documented adverse reactions associated with increased intracranial pressure. Furthermore, existing literature did not provide substantial support for the development of intracranial hypertension in patients with BD using methotrexate and thalidomide. However, there were 6 case reports involving BD combined with simple intracranial hypertension in 17 patients, which led us to consider the possibility that the elevated intracranial pressure may be a result of the BD itself rather than pharmacologic factors. Given the significantly elevated intracranial pressure observed in the patient at that time, immediate management was crucial. Therefore, methylprednisolone, azathioprine, mannitol, and methylcobalamin were administered based on the primary activity, resulting in significant alleviation of optic papilloedema (a prominent sign of elevated intracranial pressure). This finding further supports that BD in children leads to an elevation of intracranial pressure.

The limitation of this study lies in the small sample size of patients included. Therefore, further investigation with a larger sample is required to verify the aforementioned clinical characteristics. Additionally, more experience and data accumulation are necessary to establish a standardized treatment approach for NBD with isolated intracranial hypertension.

This is the first reported case of NBD with isolated intracranial pressure in pediatric patients in China. Furthermore, it marks the first literature review aimed at summarizing and analyzing the clinical characteristics of this disease. This review serves to support physicians in early diagnosis and effective treatment strategies. Even in cases where BD patients exhibit no ocular or systemic symptoms, it is imperative for them to undergo regular ophthalmological examinations. For patients with headache, diplopia/blurred vision, and vomiting, comprehensive neurological and ocular examinations should be conducted. Patients with elevated intracranial pressure should have a detailed inquiry about their medical history and physical examinations.

## Data Availability

All data analysed are included in this published article.

## Abbreviations

NBD: Neuro-Behcet's disease
BD: Behçet's disease
SL: Skin lesions
PT: Pathergy test
ST: Superficial thrombophlebitis
CSF: CerebroSpinal Fluid
GCS: Glucocorticoid
CT: Computed Tomography
MRI: Magnetic Resonance Imaging
MRA: Magnetic Resonance Angiography
MRV: Magnetic Resonance Angiography
FLAIR: Fluid attenuated inversion recovery
